# Absence of COVID-19 associated mucormycosis in a tertiary intensive care unit in the Netherlands

**DOI:** 10.1038/s41598-023-47231-4

**Published:** 2023-12-13

**Authors:** J. R. Schippers, P. E. Verweij, L. M. A. Heunks, K. van Dijk, the ArtDECO consortium, Esther J. Nossent, JanWillem Duitman, Anno Saris, Heder De Vries, Lilian J. Meijboom, Lieuwe D. J. Bos, Siebe G. Blok, Alex R. Schuurman, Tom D. Y. Reijnders, Juan J. Garcia Vallejo, Hetty Bontkes, Alexander P. J. Vlaar, W. Joost Wiersinga, René Lutter, Tom van der Poll, Harm Jan Bogaard, Leo Heunks, Shiqi Zhang, Robert F. J. Kullberg, Justin de Brabander, Leonoor S. Boers

**Affiliations:** 1 Department of Pulmonary Medicine, AmsterdamUMC, VUmc, Vrije Universiteit Amsterdam, De Boelelaan 1117, 1081 HV Amsterdam, The Netherlands; 2 Department of Medical Microbiology, Radboud University Medical Center, Nijmegen, The Netherlands; 3 Department of Intensive Care, Radboud University Medical Center, Nijmegen, The Netherlands; 4 Department of Medical Microbiology and Infection Control, AmsterdamUMC, VUMC, Vrije Universiteit Amsterdam, Amsterdam, The Netherlands; 5 Amsterdam University Medical Centers, Amsterdam, The Netherlands

**Keywords:** Respiratory distress syndrome, Outcomes research, Fungal biology

## Abstract

Mucormycosis is a severe complication in critically ill COVID-19 patients. Throughout the pandemic, a notable prevalence of mucormycosis has been observed in the Indian population, whereas lower occurrences have been reported in Europe. However, limited data exist regarding its prevalence in Europe, which is potentially underestimated due to the low sensitivity of bronchoalveolar lavage (BAL) cultures. We aimed to evaluate the prevalence of mucormycosis in a high-risk critically ill COVID-19 population in the Netherlands, and to evaluate the potential benefit of adding Mucor PCR to BAL as part of routine follow-up. In this study, we included 1035 critically ill COVID-19 patients admitted to either one of the two ICUs at AmsterdamUMC between March 2020 and May 2022; of these, 374 had undergone at least one bronchoscopy. Following the AmsterdamUMC protocols, bronchoscopies were conducted weekly until clinical improvement was achieved. We cultured BAL fluid for fungi and used PCR and galactomannan testing to detect *Aspergillus * spp. Additionally, we retrospectively performed qPCR targeting Mucorales DNA in the BAL of 89 deceased patients. All cultures were negative for Mucorales, whereas 42 (11%) cultures were positive for *Aspergillus*. Furthermore, qPCR targeting Mucorales was negative in all 89 deceased patients. This study showed that pulmonary mucormycosis was not present in critically ill COVID-19 patients in two tertiary care ICUs. These results indicate routine Mucorales qPCR screening is not clinically necessary in a high-standard-of-care tertiary ICU in a low-endemic area.

## Introduction

Patients with critically ill Coronavirus disease 2019 (COVID-19) may be prone to fungal pulmonary co-infections, which are associated with increased morbidity and mortality^1^. Risk factors for fungal infections include an impaired immune response, treatment with glucocorticosteroids, old age, and diabetes mellitus. In addition to COVID-19 associated pulmonary aspergillosis (CAPA), a high prevalence of COVID-19-associated mucormycosis (CAM) was reported in the Indian population^2,3^. In Europe, case series have indicated a much lower prevalence, which, unlike Indian cases, predominantly includes pulmonary infections in critically ill patients^4–6^. However, CAM may be underdiagnosed due to restricted sampling of the lower respiratory tract and the lack of a broadly available biomarker^7^. However, recently quantitative polymerase chain reaction (qPCR) on bronchoalveolar lavage (BAL) fluid or serum targeting Mucorales was shown to improve diagnostic sensitivity^8^. As there is currently limited data on the prevalence of CAM in European ICUs, we investigated the frequency of CAM in a high-risk population of patients with severe COVID-19 using routine BAL cultures and qPCR.

## Methods

This observational study was performed in 2 ICUs of the AmsterdamUMC in the Netherlands. Our patient cohort included all patients who had been admitted to the ICU with PCR-proven COVID-19 and had undergone at least one bronchoscopy with BAL during ICU admission. In the participating ICUs, bronchoscopy with BAL was routinely performed weekly, from endotracheal intubation until clinical improvement. Additional bronchoscopies were performed for clinical indications. All BAL fluid samples were cultured for the presence of fungi using Sabouraud dextrose agar (BD Franklin Lakes, NJ, USA; Stock-Keeping Unit 274720) supplemented with 50 mg/L chloramphenicol (Sigma, St-Louis, MO, USA; C-0378) and incubated at 30 and 37 °C. All molds were identified to the species level, using macroscopy, microscopy, and MaldiToF (Bruker Daltonik, Bremen, Germany). In addition, a subpopulation of deceased COVID-19 patients was retrospectively investigated for the presence of CAM using qPCR targeting Mucorales DNA. Of these patients, fungal DNA was extracted from the last available BAL fluid collected within two weeks before death. The BAL samples were stored at − 80 °C, with a maximum storage duration of two and a half years. DNA was extracted using the MagNAPure 96 (Roche Diagnostics, Basel, Switzerland). 300 µL lysis buffer (Roche, MagNA Pure LC Total Nucleic Acid Isolation Kit 03 246 779 001) and 20 µL of internal amplification control (in-house IAC plasmid) were added to the sample. 5 µL of eluate was added to the Mucorales qPCR. This PCR was performed using a commercial CE-IVD assay (MucorGenius®; Pathonostics, Maastricht, The Netherlands) according to the manufacturer’s instructions, with an internal amplification control, a negative control, and a positive control. The negative extraction control consisted of 300 µL of PBS, 300 µL of lysis buffer, and 20 µL of internal amplification control (in-house IAC plasmid), of which 50 µL was added to the MucorGenius®.

Additionally, data on other fungal infections (culture, galactomannan, and PCR), antifungal therapy, and glucocorticosteroid use were collected from all patients. In accordance with the 2020 ECMM/ISHAM consensus, patients presenting a positive culture, galactomannan, or PCR result in their BAL samples were categorized as having probable CAPA. If *Aspergillus* spp. was identified during the autopsy, patients were classified as proven CAPA^9^.

All patients were admitted to dedicated COVID-19 ICUs. Each patient was accommodated either alone in a room or with one other patient, and the rooms were not equipped with self-closing doors. The patient rooms were maintained at a negative pressure. Although HEPA filters were recommended, they were found to be impractical. Therefore, UF 9 air filters were employed instead. In addition, the air in the rooms was changed six times every hour. The study protocol and all study procedures were approved by the Review Committee of the AmsterdamUMC Biobank (reference 2020-065). Written informed consent was obtained from all patients or their legal representatives. This study was conducted in adherence to Good Clinical Practice guidelines and the Declaration of Helsinki.

## Results

From 06-03-2020 until 27-05-2022, 1035 patients with severe COVID-19 were admitted to one of the participating ICUs. Among these patients, 374 received at least one BAL. The fungal cultures were positive for *Aspergillus* species in 42 (11%) patients, while all cultures were negative for Mucorales.

Among the 374 patients, 89 (24%) deceased between March 2020 and August 2021 and had undergone a bronchoscopy within 2 weeks prior to death, allowing for retrospective testing with a PCR targeting Mucorales. All 89 patients had died due to respiratory failure. The median time from ICU admission to death was 19 days [15, 30]. At the time of the last BAL, 40 patients (59%) had a PaO_2_ to FiO_2_ ratio of < 100 mmHg. The last bronchoscopy was performed at a median of 5 days [4, 9] before death. 85 of 89 patients (96%) were treated with steroids during admission, the median dose per day was 42.6 mg [24.5–57.7] (prednisone equivalents), and antifungal therapy consisting of voriconazole and anidulafungin was initiated in 26 patients (29%) (Table 1). Mucorales PCR performed on BAL was negative for all 89 patients. However, *Aspergillus* spp. were detected in the BAL of 18 patients (18%). These patients were classified as probable CAPA based on positive BAL culture (3 patients), positive BAL *Aspergillus* PCR (11 patients), and positive BAL-galactomannan (7 patients)^9^. No other fungal infections were observed. An Autopsy was performed on 12 patients, all of whom had no mycological evidence of invasive fungal disease prior to death. At autopsy, no histopathological evidence of invasive fungal disease was observed in these patients. No significant differences were observed between patients with probable CAPA and non-CAPA patients in terms of admission characteristics, comorbidities, and clinical outcomes.

**Table 1:** Baseline demographics and clinical patient characteristics for the subgroup of deceased patients.

	Deceased COVID-19 patients in the ICU
Number of patients	89
Sex—Male (%)	69 (78)
Age—year [IQR]	68.5 [60.0–73.0]
BMI—kg/m^2^ [IQR]	27.3 [24.7–31.2]
Smoking—Yes/Former (%)	30 (57)
Asthma—Yes (%)	6 (7)
COPD—Yes (%)	4 (5)
Interstitial lung disease—Yes (%)	1 (1)
Active solid malignancy—Yes (%)	5 (6)
Active hematological malignancy—Yes (%)	10 (11)
Diabetes—Yes (%)	29 (33)
Probable CAPA—Yes (%)	16 (18)
EORTC host factor—Yes (%)	32 (36)
Number of EORTC host factors (%)	
0	57 (64)
1	17 (19)
2	7 (8)
3	6 (7)
4	2 (2)
Cumulative amount of corticosteroids—mg [IQR]	500 [400–1172]
Median amount of steroids per day—mg [IQR]	42.6 [24.5–57.7]
IL-6 receptor inhibitor—Yes (%)	10 (11)
Antifungal therapy—Yes (%)	26 (29)
Duration antifungal therapy—Days [IQR]	4 [3–8]
Time in ICU—Days [IQR]	19 [15–30]
SOFA score [IQR]	8 [7–10]
CT severity score [IQR]	24 [21–25]
PaO_2_/FiO_2_ ratio—mmHg [IQR]	92.7 [70.5–117.3]
Number of BAL per patient—median [IQR]	2 [1, 2]
Mono organ failure—Yes (%)	47 (53)
Autopsy—Yes (%)	12 (14)
Time between BAL and death—Days [IQR]	5 [4–9]

Continuous data are described as median with interquartile range.

BMI, body mass index; BAL , Broncho-alveolar lavage; CAPA, COVID-19 Associated Pulmonary Aspergillosis; COPD, chronic obstructive pulmonary disease; CT, computed tomography; EORTC, European Organization for Research and Treatment of Cancer; ICU, intensive care unit; IL-6, Interleukin-6; IQR, inter quartile range; SOFA, sequential organ failure assessment.

## Discussion

**Figure 1:**
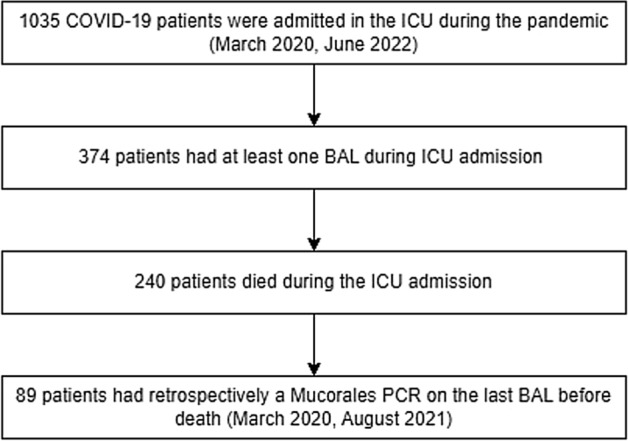
Diagram of the patient flow. BAL, Broncho-Alveolar Lavage; COVID-19, Coronavirus Disease 2019; ICU, intensive care unit; PCR, polymerase chain reaction.

In our cohort of 374 patients, having received at least one BAL, no cases of CAM were identified, corresponding to a prevalence of 0% (95% CI: 0–0.010%, Fig. 1). Our cohort included patients that had undergone routine weekly bronchoscopy. Furthermore, in addition to BAL fungal culture, we used Mucorales PCR in a subgroup of patients who died due to respiratory failure. This approach limits the possibility of missing CAM cases; however still, no cases were identified. Risk factors for CAM include diabetes mellitus and corticosteroid use, which were also predominant in our subgroup (Table 1). Furthermore, the CAPA incidence of 18% indicates that our cohort with critically ill COVID-19 was prone to developing secondary fungal infections^10^.

A case series from European countries, including the Netherlands, indicated that CAM may occur in COVID-19 patients, but an accurate estimate of the prevalence could not be given as a denominator is unknown^4–6^. The baseline epidemiology of mucormycosis is not well studied, but a population-based study from France indicated an incidence of 0.09 cases per 100,000 population per year, prior to the corona pandemic^11^. For the ICU population, no frequency estimates are available for mucormycosis^12^; however, in critically ill patients with COVID-19, a CAM prevalence of 1% has been reported^4^. Our study confirms a very low prevalence of CAM in the ICU despite the presence of risk factors for fungal infections in the majority of patients and intensive sampling.

Strengths of this study were the intensive sampling and the additional use of qPCR, which improved the diagnostic value of culture alone. Recovery of Mucorales through the culture of clinical specimens may be difficult, and the use of qPCR improves diagnostic sensitivity^8^. Nonetheless, CAM cases were not observed. A limitation of this study was that serum samples were not available for qPCR testing^13^. However, all patients died primarily because of respiratory failure; therefore, the lungs were expected to be the primary site for CAM. Another limitation is the lack of autopsies in the group of 89 patients receiving a qPCR.

In conclusion, this study showed that pulmonary CAM was not present in critically ill COVID-19 patients in two tertiary care ICUs. These results indicate that there is no clinical need for routine screening with qPCR targeting Mucorales in a high-standard-of-care tertiary ICU in a low-endemic area.

## Data Availability

The datasets used and/or analyzed in the current study are available from the corresponding author upon reasonable request.
